# Treatment of femoral shaft fractures with monoaxial external fixation in polytrauma patients

**DOI:** 10.12688/f1000research.11893.1

**Published:** 2017-08-07

**Authors:** Gianluca Testa, Domenico Aloj, Alessandro Ghirri, Eraclite Petruccelli, Vito Pavone, Alessandro Massé

**Affiliations:** 1 Department of Orthopaedics and Traumatologic Surgery, AOU Policlinico-Vittorio Emanuele, University of Catania, Catania, Italy; 2 Department of Traumatology, PO Sant’Andrea, Vercelli, Italy; 3 Department of Orthopaedics Surgery, Division of Muscular-Skeletal Traumatology, AOU Città della Salute, CTO Hospital, Turin, Italy

**Keywords:** femoral shaft fractures, polytrauma, monoaxial external fixator, definitive treatment

## Abstract

**Background**: Femoral shaft fractures, typical in younger people, are often associated with polytrauma followed by traumatic shock. In these situations, despite intramedullary nailing being the treatment of choice, external fixation could be used as the definitive treatment. The aim of this study is to report evidence regarding definitive treatment of femoral shaft fractures with monoaxial external fixation.

**Methods**: Between January 2006 and December 2015, 83 patients with 87 fractures were treated at the Department of Orthopaedics and Traumatology CTO of Turin, with a monoaxial external fixation device. Mean age at surgery, type of fracture, mean follow-up, time and modalities of treatment, non-weight bearing period, average healing, external fixation removal time, and complications were reported.

**Results**: The average patient age was 31.43±15.19 years. In 37 cases (42.53%) the right femur was involved. 73 (83.91%) fractures were closed, and 14 (16.09%) were open. The average follow-up time was 61.07±21.86 weeks.  In 68 (78.16%) fractures the fixation was carried out in the first 24 hours, using a monoaxial external fixator. In the remaining 19 cases, the average delay was 6.80±4.54 days. Mean non-weight bearing time was 25.82±27.66 days (ranging from 0 to 120). The 87 fractures united at an average of 23.60±11.37 weeks (ranging from 13 to 102). The external fixator was removed after an average of 33.99±14.33 weeks (ranging from 20 to 120). Reported complications included 9.19% of delayed union, 1.15% of septic non-union, 5.75% of malunion, and 8.05% cases of loss of reduction.

**Conclusions**: External fixation of femoral shaft fractures in polytrauma is an ideal method for definitive fracture stabilization, with minimal additional operative trauma and an acceptable complication rate.

## Introduction

Femoral shaft fractures are typical in younger people ^1^, and can be caused by car accidents, falling down from heights or gunshot wounds ^2^. Several intensive traumatic agents frequently bring about comminuted and open femoral shaft fractures ^3^. These fractures are typically associated with polytrauma, followed by traumatic shock ^4^.

Intramedullary nailing is considered to be the treatment of choice for fixation of most femoral shaft fractures ^5– 7^. However, there are instances where fixation with intramedullary nailing cannot not be performed, for example during severe polytrauma, when the general condition of patients precludes major surgery and there are severe open fractures with extensive soft tissue damage. In these situations, external fixation is used for temporary fixation. Surgical conversion from external fixation to intramedullary nailing within one to two weeks of the injury is the standard practice ^8^; however, due to financial constraints, in large parts of the world external fixation of femoral shaft fractures is often the definitive treatment ^9^.

The aim of this study is to report monoaxial external fixation as the definitive treatment of femoral shaft fractures.

## Methods

Between January 2006 and December 2015, 160 patients with 182 femoral shaft fractures were treated at the Department of Orthopaedics and Traumatology CTO of Turin, with monoaxial external fixation, Orthofix Procallus®. The study was conducted according to the principles expressed in the Declaration of Helsinki. Only fractures with external fixation as the definitive treatment were included. Patients who did attend follow-ups or who died for reasons unrelated to the fracture, such as cardiopulmonary arrest or septicaemia, were ruled out.

Data on 83 patients with 87 fractures were gathered retrospectively, from hospital records. Follow-ups were carried out for a minimum period of 39 weeks (9 months), or until bone union. The reasons for injury were motor vehicle accidents in all cases.

Age at surgery, gender, injured side, location and type of fracture, AO classification, mean follow-up time and modalities of treatment, non-weight bearing period time, average union time, and external fixation removal time were recorded. Bone union was clinically and radiographically evaluated, according to common criteria in the literature. At clinical assessment, fractures were considered healed, when the absence of movement and pain on stress at the fracture site was observed. Radiographic union was achieved in the presence of uniform and continuous ossification of callus, with consolidation and development of trabeculae across the fracture site ^10^.

Union time of more than 26 weeks in closed fractures and 39 weeks in open fractures was considered a delayed union ^11– 13^. The diagnosis of non-union was made in the presence of abnormal movement at the fracture site at least 9 months after the injury and with no progressive signs of healing for at least 3 months, despite continuing treatment ^12^. Malunion was defined with one of the following criteria: shortening of more than 2.5 cm, angulation of more than 10°, or rotational malalignment of more than 5°. Major and minor complications with secondary surgical procedures were noted.

## Results

The average patient age was 31.43±15.19 years (ranging from 14 to 87). There were 66 men (79.52%) and 17 women (20.48%). Four patients (4.82%) had a bilateral femur fracture. In 37 cases (42.53%) the right femur was involved, and in 50 cases (57.47%) the left femur. In 14 cases (16.09%) the fracture was located in the proximal third of the femur, in 57 cases (65.52%) in the middle third of the femur and in 16 cases (18.39%) in the distal third. 73 fractures (83.91%) were closed, and 14 (16.09%) were open ( Table 1). Following the AO classification of fractures, there were: 4 (4,60%) 32A1, 13 (14,94%) 32A2, 21 (24,14%), 32A3, 7 (8.05%) 32B1, 11 (12.64%) 32B2, 12 (13.79%) 32B3, 3 (3.45%) 32C1, 3 (3.45%) 32C2, and 13 (14,94%) 32C3 ( Table 2). Of 14 open fractures, following Gustilo-Anderson classification, there were: 6 GI (42.86%), 4 GII (28.57%), 1 GIIIa (7.14%), 2 GIIIb (14.29%), and 1 GIIIc (7.14%) ( Table 3).

**Table 1.** Classification of fractures by location.

	*Proximale*	*Middle*	*Distal*	*Total*
*N*	14	57	16	87
*Rate*	16.09%	65.52%	18.39%	100%

**Table 2.** Classification of fractures by AO criteria.

	*32A1*	*32A2*	*32A3*	*32B1*	*32B2*	*32B3*	*32C1*	*32C2*	*32C3*	*Tot.*
*N*	4	13	21	7	11	12	3	3	13	87
*%*	4.60	14.94	24.14	8.05	12.64	13.79	3.45	3.45	14.94	100

**Table 3.** Classification of fractures by level of exposure.

	*Closed*	*GI*	*GII*	*GIIIa*	*GIIIb*	*GIIIc*
*N°*	73	6	4	1	2	1
*Rate*	83.90%	42.86%	28.57%	7.14%	14.29%	7.14%

The average follow-up time was 61.07±21.86 weeks (ranging from 28 to 160). In 68 fractures (78.16%) the fixation was carried out in the first 24 hours, using a monoaxial external fixator. In the remaining 19 cases, the average delay was 6.80±4.54 days (ranging from 3 to 20). Of these 19 patients, 7 (8.05%) had skeletal traction and 12 (13.79%) a stabilization with temporary external fixation. Mean surgery duration time was 55.36±11.13 minutes (ranging from 35 to 80).

The patients were mobilized with crutches as soon as possible, with a gradual increase of weight bearing within tolerable limits of pain. Weight bearing was not immediately allowed in patients with other associated lower limb fractures or severe systemic complications. Mean non-weight bearing time was 25.82±27.66 days (ranging from 0 to 120).

**Figure 1.**
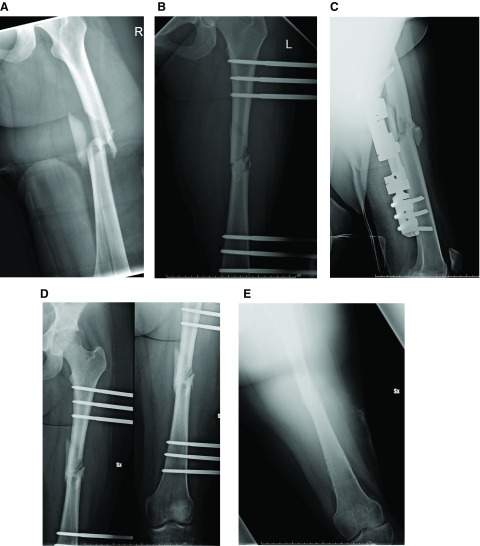
**A**. Femoral shaft fracture in a patient, AO type 32C3. **B**. Post-operative radiographic evaluation in AP view. **C**. Post-operative radiographic evaluation in lateral view. **D**. Radiographic evaluation after three months from surgery. **E**. External fixator removal after 7 months from surgery.

The 87 fractures united at an average of 23.60±11.37 weeks (ranging from 13 to 102). The external fixator was removed after sufficient callus was seen at an average of 33.99±14.33 weeks (ranging from 20 to 120) ( Table 4). Please see Figure 1 to see the progression of a patient treated with external fixation after a femoral shaft fracture.

**Table 4.** Results of the patient details that were recorded.

	*Time*
***Average follow-up (weeks)***	61.07±21.86 (range 28–160)
***Mean surgery duration time (min.)***	55.36±11.13 (range 38–80)
***Mean fixator removal time (weeks)***	33.99±14.33 (range 20–120)
***No weight-bearing time (days)***	25.82±27.66 (range 0–120)
***Healing time (weeks)***	23.60±11,37 (range 13–102)

Excluding the delayed unions, 79 (90.8%) fractures united at an average time of 20.80±3.59 weeks (ranging from 13–30 weeks). Eight fractures (9.2%) had delayed union, with an average union time of 51.25±21.97 weeks (ranging from 36–102 weeks). All of the delayed unions occurred in closed comminuted fractures, with or without bone loss in multiply injured patients. Bone loss of 5 cm or more was noted in 9 patients (10.34%) ( Table 5).

**Table 5.** Average femoral shaft fracture union time.

	*All fractures*	*Union*	*Delayed union*
*N*	87	79	8
*Average* *union* *(weeks)*	23.60±11.37 (range 13–102)	20.08±3.59 (range 13–30)	51.25±21.97 (range 26–102)

Secondary surgical procedures were performed in eight cases of delayed union (9.19%): 2 corticocancellous grafts 12 months after injury; one fibular graft 11 months after injury; 2 applications of ring fixator 8 months after injury; 2 applications of circular hexapod external fixator 8 months after injury; and 1 reduction and fixation precedure with plates and screws after 15 months. Septic non-union occurred in two fractures (2.3%), and treatment involved surgical debridement and application of a ring fixator. Malunion occurred in five (5.75%) cases: two shortenings of 3 cm and one varus deformity corrected with application of ring external fixator; two recurvatum deformity associated internal rotation deformity of 20°, treated with hexapod external fixator. Two re-fractures occurred (2.3%), which were successfully treated with repeat monoaxial external fixation.

Loss of reduction after external fixation was observed in seven cases (8.05%) and treated with an external fixator reset.

One major complication, a decrease in the range of motion of the knee, occurred in one patient (1.15%). The fracture was located in the distal third of the femur. In this case a Judet arthromiolysis ^14, 15^ was performed.

Minor complications, namely pin-tract infections, were noted in 12 (13.8%) cases but did not influence the outcome; they were managed by improvement of hygiene and antibiotic therapy. Breakage of Schanz screws was reported in one case (1.15%) and successfully managed by debridement and removal and re-insertion of the screw. One patient (1.15%) had pain at the fracture location after removal of the external fixator, so it was repositioned for another 2 months ( Table 6).

**Table 6.** Rate of complications of femoral external fixation in this series.

	*N*	*Rate*
*Delayed union*	8	9.2%
*Septic non-union*	2	2.3%
*Malunion*	5	5.75%
*Refracture*	2	2.30%
*Loss of reduction*	7	8.05%
*Knee joint stiffness*	1	1.15%
*Pin tract infections*	12	13.8%
*Breakage of screws*	1	1.15%
*Painful thigh*	1	1.15%

Data and details of the 83 patients that underwent treatment for femoral shaft fractures, used as a basis for the findings in this study Click here for additional data file. Copyright: © 2017 Testa G et al. 2017 Data associated with the article are available under the terms of the Creative Commons Zero "No rights reserved" data waiver (CC0 1.0 Public domain dedication).

## Discussion

Intramedullary nailing for the treatment of femoral shaft fractures was introduced by Groves in United Kingdom and Kuntcher in Germany ^16– 18^. Today, reduction and fixation with reamed intramedullary nailing is considered the gold standard for the treatment of most femoral shaft fractures ^5– 7^.

External fixation is not widely used for femoral shaft fractures, and there are few studies in the literature that have reported this use. External fixation has been generally reserved for initial stabilization of polytrauma patients, or for open fractures ^19^. Early stabilization in polytrauma patients could decrease morbidity and mortality, avoiding pulmonary complications, including pneumonia, fat embolism and acute respiratory failure ^20, 21^, although a delay of surgery up to 72 hours does not increase the risk of complications ^22^. The reported benefits included improved patient mobility, improvement of pulmonary hygiene, decreased pain and reduced need of narcotics ^23^. Moreover, the procedure is rapid and could be performed in around 30 minutes, even though in our study the mean duration was 55 minutes, because more time was needed to treat soft tissue and skin. This is particularly important for patients in critical condition, and in cases of open fractures with relevant damages to the vascular supply of the bone ^24^.

Open fractures are usually associated with severe comminution at the fracture site and bone loss ^25^, so external fixation is the treatment of choice because it stabilizes the fracture and allows any soft-tissue wound to be treated daily, as necessary ^19^. In these cases, unlike internal fixation devices ^24 ^, external fixation spares uninjured tissue planes and the periosteal circulation, allowing vascular repair ^26^. Using the data collected for our study, only 14 (16.09%) fractures were open, with more than 50% of II, IIIa, IIIb, IIIc types. The treatment of such fractures was associated with increased risk of infection and delayed union ^27^.

We applied the concept of damage control surgery, based on management of multiply-injured patients with associated fractures of long bones and pelvic fractures. This concept consists of an early temporary stabilization of unstable fractures, control of haemorrhage and treatment of possible abdominal or intracranial lesions. When the condition of the patient has been optimized, it is possible to perform a delayed definitive management of fractures. The delayed, definitive stabilization procedure of femoral fractures that has been most commonly used, was the removal of the external fixation and intramedullary nailing of the fracture ^28^. In our series, we performed definitive external fixation within 24 hours in 78% of cases, while in the remaining 22% skeletal traction or temporary external fixation was performed.

Mean healing time in our study of femoral fractures treated by external fixation was 23.60 weeks (ranging from 13 to 102), similar to previous studies ^9, 19, 29, 30^. In most reported cases, patients had been given some form of after-support (braces, casts) after approximately 3 to 7 months ^8, 26^. In our series, removal of external fixation was performed at an average time of 34 weeks, with application of brace until clinical stability of fracture.

Main complications reported in the literature were pin-tract infections and contracture of the knee joint ^29, 30^, the risk of these happening can be minimized with good pin hygiene, antibiotic therapy and knee exercises ^29^. Pin-tract infections was registered in 13.8% of cases in our study, while there was only one severe contracture of the knee joint, treated with Judet arthromiolysis ^15^.

Other complications, such as delayed union and re-fractures, were successfully resolved with secondary surgical procedures, such as corticocancellous grafts, or applications of a ring fixator ^9^. In one case, a fibular graft was necessary. Malunion was typically treated by changing the external fixation. Only in one case the external fixation was removed and an internal fixation device applied to correct a recurrent valgus deformation.

Although the external fixation is considered a safe procedure to achieve temporary rigid stabilization in patients with multiple injuries at risk of an adverse outcome ^8^, we performed external fixation as definitive management, because for patients with polytrauma, we preferred to avoid another surgical procedure such as a conversion to an internal device. Indeed, our rate of septic nonunion was 2.3%, which is comparable to the rate of seen with intramedullary nailing ^8, 19^. Septic nonunion was managed with surgical debridement and application of a ring fixator.

In conclusion, external fixation of femoral shaft fractures in polytrauma patients is an ideal method of fracture stabilization, with minimal additional operative trauma. Satisfactory outcomes can be reported using a damage control strategy for these fractures, before definitive external fixation, with acceptable complication rates and a reduced need of other open and invasive surgical procedures. A strict postoperative protocol, including early weight-bearing, intensive physical therapy and protection of the bone after complete removal, needs to be followed. Pin tract infections are the main complications and can be treated by local wound care and antibiotic therapy.

## Data availability

The data referenced by this article are under copyright with the following copyright statement: Copyright: © 2017 Testa G et al.

Data associated with the article are available under the terms of the Creative Commons Zero "No rights reserved" data waiver (CC0 1.0 Public domain dedication). 

Dataset 1: Data and details of the 83 patients that underwent treatment for femoral shaft fractures, used as a basis for the findings in this study. DOI, 10.5256/f1000research.11893.d170645 ^31^

## Ethical statement

This study has been conducted according to the principles expressed in the Declaration of Helsinki.

Ethical approval was not necessary in this study because the data and clinical pictures have been sufficiently anonymised. Written informed consent for anonymous publication of their clinical details and clinical images was obtained from all patients. The Department-Chief, Alessandro Massé, authorized the authors to take information about patient records, allowing their use for this study. CTO Hospital of Turin owns the patient data that was recorded for Gianluca Testa and Alessandro Ghirri.
