# Supplementary material for: Treatment of femoral shaft fractures with monoaxial external fixation in polytrauma patients
Source: F1000Res. 2017 Aug 7;6:1333. [Version 1] doi: 10.12688/f1000research.11893.1 (PMC5580418; doi:10.12688/f1000research.11893.1)
Supplement: Peer review history

# Peer review: Referee response for version 1

**DOI:** 10.5256/f1000research.12851.r24820

**Published:** 2017-08-31

**Authors:** Saverio Comitini

**Affiliations:** 1 Dipartimento di Ortopedia e Traumatologia, Ospedale Maggiore, Bologna, Italy

The manuscript treated about definitive monoaxial external fixation for shaft femural fractures in polytrauma cases. I agree with the choice of Authors for this option of treatment, useful to minimize the surgical duration time and blood loss. External fixation technique is easy and reproducible, but associated with minor complications, as superficial pin infections. This is the reason why surgeons are not encouraged to use this device as definitive solution, and also for the poor attitude to allow an early-weight bearing.

I have read this submission. I believe that I have an appropriate level of expertise to confirm that it is of an acceptable scientific standard.

# Peer review: Referee response for version 1

**DOI:** 10.5256/f1000research.12851.r24818

**Published:** 2017-08-29

**Authors:** Lorenza Marengo

**Affiliations:** 1 Department of Pediatric Orthopedic and Traumatology, "Cesare Arrigo" Children’s Hospital, Alessandria, Italy

This is a well written manuscript on femoral shaft fracture in polytrauma patients treated by monoaxial external fixation as definitive treatment. The sample characteristic are adequate and the study is well designed, I suggest to define more precisely the amount of loss of range of motion when considered as a complication as well as when arhrtomyolisis was indicated.

 Furthermore, it could be interesting to compare the average duration of the surgical procedure in closed and open fracture groups. The reported duration for external fixator application as initial stabilization device for femoral shaft fractures in polytrauma patients is around 30 minutes. In this study, the average duration is higher (55 minutes). The authors of the study suggested that this difference in duration is explained by the fact that “more time is needed to treat soft tissue and skin”. However, the difference might be the result of using the external fixation as definitive treatment, which requires better reduction and more stable construct. If this is the case, the surgical time might increase also for closed fracture group.

 In conclusion, this manuscript reported external fixation to be a valid option as definitive treatment for femoral shaft fracture in polytrauma patients. This technique allows early fracture stabilization and eliminate the need of additional surgery for obtaining definitive fracture reduction and stabilization in 78% of the patients.

 The topic of this manuscript is interesting and it deserves serious consideration for publication.

I have read this submission. I believe that I have an appropriate level of expertise to confirm that it is of an acceptable scientific standard.

# Peer review: Referee response for version 1

**DOI:** 10.5256/f1000research.12851.r24819

**Published:** 2017-08-10

**Authors:** Dario Pitino

**Affiliations:** 1 Shoulder and Elbow Surgery Unit, Humanitas Research Hospital, Rozzano, Italy

I suppose this choice was due to critical conditions of patients. My suggestion is to specify this concept in the materials and methods. This may explicate also the validity of this kind of definitive treatment, associated with high rate of complications.

 I consider the manuscript valid and suitable for publication.

I have read this submission. I believe that I have an appropriate level of expertise to confirm that it is of an acceptable scientific standard.
